# Unruptured tubal pregnancy: different treatments for early and late diagnosis

**DOI:** 10.1590/S1516-31802006000600004

**Published:** 2006-11-01

**Authors:** Julio Elito, Luiz Camano

**Keywords:** Ectopic pregnancy, Methotrexate, Chorionic gonadotropin, Ultrasonography, Amenorrhea, Gravidez ectópica, Metotrexato, Gonadotropina coriônica, Ultrasonografia, Amenorréia

## Abstract

**CONTEXT AND OBJECTIVE:**

There is evidence that ectopic pregnancies present different behavioral patterns. These distinct evolutions of ectopic pregnancies represent two different natural histories. To evaluate these evolution patterns, we compared patients undergoing medical treatment and expectant management according to their gestational age and initial beta-hCG levels.

**DESIGN AND SETTING:**

Prospective study at the Department of Obstetrics of Universidade Federal de São Paulo, a tertiary center.

**METHODS:**

Among 119 cases of unruptured ectopic pregnancies diagnosed from April 1999 to February 2004, 57 received systemic treatment with methotrexate 50 mg/m2 (body surface area) intramuscularly and 62 were managed expectantly. In this study we evaluated the beta-hCG levels at presentation and amenorrhea duration.

**RESULTS:**

There was a significant difference between the two groups regarding amenorrhea duration and initial beta-hCG levels (p < 0.001). The group with decreasing beta-hCG levels (managed expectantly) had longer amenorrhea (mean: 8.87 ± 1.71 weeks) and lower initial beta-hCG levels (mean: 648.8 ± 754.7 mIU/ml). On the other hand, the group treated with methotrexate had shorter amenorrhea (mean: 6.81 ± 1.88 weeks) and higher beta-hCG levels at presentation (2642.7 ± 2315.1 mIU/ml).

**CONCLUSIONS:**

The data suggest that ectopic pregnancies can be categorized into two groups: those with early diagnosis and shorter amenorrhea, increasing or maintained beta-hCG levels over 24 and 48-hour intervals and higher beta-hCG levels requiring medical treatment; and those with late diagnosis and longer amenorrhea, decreased beta-hCG levels over 24 and 48-hour intervals and lower beta-hCG levels requiring expectant management.

## RESUMO

**CONTEXTO E OBJETIVO:**

A gravidez ectópica apresenta comportamentos diferentes. Para avaliar as diferentes evoluções da gravidez ectópica, nós comparamos as pacientes submetidas ao tratamento medicamentoso e à conduta expectante de acordo com a idade gestacional e os valores iniciais de beta-hCG.

**TIPO DE ESTUDO E LOCAL:**

Estudo prospectivo, realizado no Departamento de Obstetrícia da Universidade Federal de São Paulo, hospital terciário.

**MÉTODOS:**

Foram diagnosticados 119 casos de gravidez ectópica íntegra no período de abril de 1999 a fevereiro de 2004. O tratamento com metotrexato 50 mg/m^2^ de superfície corpórea intramuscular foi realizado em 57 casos e a conduta expectante, em 62 casos. Neste estudo, nós avaliamos os valores iniciais de beta-hCG e o período de amenorréia.

**RESULTADOS:**

Houve diferença estatística significante entre os dois grupos com relação ao período de amenorréia e os valores iniciais de beta-hCG (p < 0,001). O grupo com títulos de beta-hCG em regressão (conduta expectante) apresentaram período de amenorréia mais longo (média:8,87 ± 1,71 semanas) e valores iniciais de beta-hCG mais baixos (648,8 + 754,7 mUI/ml). Por outro lado, o grupo tratado com metotrexato teve período menor de amenorréia (média 6,81 + 1,88 semanas) e valores de beta-hCG mais altos (2642,7 +2315,1 mUI/ml).

**CONCLUSÕES:**

Os resultados deste estudo sugerem que a gravidez ectópica pode ser categorizada em dois grupos: aquelas com diagnóstico precoce com período curto de amenorréia e valores elevados de beta-hCG que requerem tratamento com metotrexato, e aquelas com diagnóstico tardio com período longo de amenorréia e valores baixos de beta-hCG que requerem a conduta expectante.

## INTRODUCTION

The prevalence of ectopic pregnancy has increased over the last few years and accounts for 2% of all pregnancies in the United States.^1^ Although this is mostly attributable to the increasing prevalence of fallopian tube disease, it is partially explained by the detection of ectopic pregnancies that would have remained undetected and would have spontaneously resolved. More accurate detection of ectopic pregnancy has been made possible by the association of the beta subunit of human chorionic gonadotropin (beta-hCG) and transvaginal ultrasonography.^2-4^

The diagnosing of ectopic pregnancy consistently leads to two different situations.^5,6^ Approximately half of the women with ectopic pregnancy are diagnosed at the time of their initial presentation and the other half are diagnosed only after further medical surveillance.^6^ This clinical scenario may represent two hypothetical natural histories for women with ectopic pregnancy: acute (early diagnosis) and chronic (late diagnosis). Some acute cases present rapid evolution of the ectopic pregnancy, and the rupturing of the tubal pregnancy demonstrates the risk in this disease. It is the leading pregnancy-related cause of death during the first trimester of pregnancy and a major contributor towards maternal morbidity.^7^ On the other hand, some chronic cases present spontaneous resolution of the ectopic pregnancy.

When a patient has an ectopic pregnancy, initially the beta-hCG levels increase until a critical point, and two events may occur. Either the tube ruptures because of erosion caused by the trophoblast tissue, or the organism may reabsorb the ectopic pregnancy and spontaneously resolve the problem. These distinct evolutions of the ectopic pregnancy represent two different natural histories. These two hypothetically distinct natural histories for ectopic pregnancy may be demonstrated by the evolution of the beta-hCG titers and the duration of amenorrhea. In the cases where the beta-hCG levels increase (early diagnosis), there is a risk of tubal rupture if no treatment is performed. On the other hand, if the betahCG levels decrease (late diagnosis), the risk of rupture has possibly passed by and, in these cases, no treatment is necessary since it may resolve spontaneously.

## OBJECTIVE

The purpose of this study was to compare a group of patients with ectopic pregnancy undergoing medical treatment with a single dose of methotrexate (50 mg/m^2^ of body surface area) intramuscularly with a group of patients undergoing expectant management. In this study, the beta-hCG levels at presentation and the amenorrhea duration were evaluated.

## MATERIALS AND METHODS

This prospective study was performed at the Department of Obstetrics of Escola Paulista de Medicina, Universidade Federal de São Paulo, from April 1999 to February 2004. During this period 185 cases of unruptured ectopic pregnancy were diagnosed: 33 underwent surgery; 65 received systemic treatment with methotrexate 50 mg/m^2^ (body surface area) intramuscularly; and 87 were managed expectantly. We excluded 25 cases of expectant management and eight cases of use of methotrexate from our sample because these patients were unsure about when their last menstrual period was.

The study was approved by the institution's ethics committee. A written letter of consent, in which the entire procedure was described, was signed by all patients.

Ectopic pregnancies were diagnosed by evaluation of the patient's clinical history (pain, bleeding and amenorrhea), gynecological examination, transvaginal ultrasonography, and measurement of serum beta-hCG levels. After confirmation of the diagnosis (i.e. the presence of an extraovarian adnexal mass ≤ 3.5 cm), further two beta-hCG titers were obtained 24 and 48 hours later.

Serum beta-hCG concentrations were measured using an enzyme immunoassay kit (AIA-pack beta-hCG, Tosoh Medic, Inc., Yamaguchi, Japan), in accordance with the Third International Standard. Transvaginal ultrasonography was carried out using a 7.5-MHz transvaginal probe with Synerg equipment (General Electric, Milwaukee, USA).

The criterion for ultrasound confirmation was the presence of an extraovarian adnexal mass in patients with a suspected ectopic pregnancy (amenorrhea, bleeding and pain) who tested positive for beta-hCG. The rule in performing the ultrasound e xamination was first to evaluate the uterine cavity to exclude an entopic pregnancy. The ovaries then had to be identified and checked for the presence of a corpus luteum. Finally, the vicinity of an ovary with a corpus luteum had to be searched for an extraovarian adnexal mass (live embryo or tubal ring or hematosalpinx), since ectopic pregnancies are mostly on the same side as the corpus luteum. If an extraovarian adnexal mass was not seen on the same side as the corpus luteum, the other side had to be searched. When the ultrasound examination was performed in this sequence, the diagnosis of ectopic pregnancy was confirmed in all the cases in this study.

The inclusion criteria for methotrexate treatment were hemodynamic stability, serum beta-hCG levels increasing or maintained over 24 and 48-hour intervals, an extraova rian adnexal mass of 3.5 cm or less in diameter, the patient's desire for a future pregnancy, and the patient's consent to participate in the study, obtained by means of signing a document that had been approved by the institution's ethics committee.^8-11^

Clinically unstable patients who had a history of sensitivity to methotrexate, or who had blood dyscrasia, liver diseases or kidney diseases, were excluded.

Patients satisfying the selection criteria were treated with a single intramuscular metho trexate dose of 50 mg/m^2^ (body surface area). During the follow-up period we obtained beta-hCG levels one, four and seven days after methotrexate injection. The protocol stipulated that any patient who did not have a decline of 15% in betahCG levels between days four and seven would be given a second intramuscular methotrexate dose of 50 mg/m^2^ one week after the first dose. Patients with declining beta-hCG levels between days four and seven were monitored as outpatients weekly until their beta-hCG levels were below 5 mIU/ml. Hospitalization was indicated for women with significant abdominal pain or suspected tubal rupture.^8,9^

The treatment was considered successful when the beta-hCG levels went down to below 5 mIU/ml, and was considered to have failed when surgery became necessary.

The inclusion criteria for expectant management were the same as the criteria for methotrexate treatment.

In all cases, the diagnosis was confirmed by the characteristic ultrasound image of an extraovarian adnexal mass.

The patients were monitored weekly until the beta-hCG levels were below 5 mIU/ml.

For statistical analysis, the Mann-Whitney test was performed for two independent samples, with regard to the time interval since the last menstrual period and initial beta-hCG levels at presentation. *p-*values of less than 0.05 were considered statistically significant.

## RESULTS

An extraovarian adnexal mass was identified in every patient of this study. Out of 119 patients with unruptured ectopic pregnancy, 62 had decreasing beta-hCG levels and were managed expectantly and 57 had serum beta-hCG levels that increased or were maintained over 24 and 48-hour intervals and were treated with methotrexate (50 mg/m^2^ intramuscularly). The success rate in the methotrexate group was 75.4% and in expectant management 95.4%.

The group managed expectantly and the group treated with methotrexate (50 mg/m^2^ intramuscularly) were compared by means of univariate analysis (Tables 1 and 2). There were significant differences between the two groups with regard to the time interval since the last menstrual period and the initial beta-hCG levels (p < 0.001). The group with decreasing beta-hCG levels (managed expectantly) had longer intervals since the last menstrual period (mean: 8.87 ± 1.71 weeks) and lower initial beta-hCG levels (mean: 648.8 ± 754.7 mIU/ml). On the other hand, the group treated with methotrexate had shorter intervals since the last menstrual period (mean: 6.81 ± 1.88 weeks) and higher beta-hCG levels at presentation (2642.7 ± 2315.1 mIU/ml).

**Table 1:** Comparison between the group managed expectantly and the group treated with methotrexate, in relation to the length of time since the last menstrual period

Last menstrual period	n	Mean ± SD
Managed expectantly	62	8.87 ± 1.71
Methotrexate	57	6.81 ± 1.88

*p < 0.001; SD = standard deviation*.

**Table 2:** Comparison between the group managed expectantly and the group treated with methotrexate, in relation to the initial beta-hCG levels

Last menstrual period	n	Mean ± SD
Managed expectantly	62	648.8 ± 754.7
Methotrexate	57	2642.7 ± 2315.1

*p < 0.001; SD = standard deviation*.

## DISCUSSION

Ectopic pregnancy is a challenge for the gynecologist. It is associated with life-threatening risk and is considered to be a dangerous disease. Fear of tubal rupture caused by the uncertainty of this clinical situation induces gynecologists to take rapid decisions to solve the problem. However, knowledge of this disease has demonstrated that patients present a broad spectrum of symptoms. Thus, ectopic pregnancy does not always end in tubal rupture. Even without intervention, ectopic pregnancies may abort from their implantation with minimal bleeding or may be reabsorbed.

Some women have aggressive ectopic pregnancies that are clearly visible on ultrasound, with high beta-hCG levels, short amenorrhea duration and acute clinical courses. However, other women have latent ectopic pregnancy with low beta-hCG levels, and longer time intervals since the last menstrual period, and they present few symptoms.^12^

During the first six weeks of amenorrhea, with methotrexate, in relation to the initial beta-hCG levels serum beta-hCG increases exponentially, i.e. when the logarithm of beta-hCG concentration is plotted against gestational age, a straight line is obtained. This means that the time taken for the beta-hCG level to double during the first six weeks is relatively constant, regardless of the initial level, in intrauterine pregnancy. In screening for ectopic pregnancy, serial beta-hCG measurements are important when the levels are less than 2000 mIU/ml and when transvaginal ultrasound is used to rule out intrauterine pregnancy.^2^

Initially, patients with ectopic pregnancies may have a 50-66% increase in betahCG levels every two days^2^ until a critical point at which two events may occur: tubal rupture or spontaneous resolution. The explanation for these events is proba bly related to the aggressiveness of the trophoblast tissue. Less aggressive invasion of the trophoblast tissue is associated with spontaneous resolution. On the other hand, more aggressive invasion is related to tubal rupture. There are probably several explanations for the intensity of aggression of the trophoblast tissue, such as immunological response and embryonic factors.^12^

Patients with ectopic pregnancies usually have a broad spectrum of symptoms and physicians need to consider several criteria in order to decide on the type of treatment. If the beta-hCG titers increase, patients have to be treated, or else they may suffer ruptured ectopic pregnancy. On the other hand, for patients at a more advanced stage, with greater gestational age and declining beta-hCG titers, the risk of rupture is almost over. In this case, the physician can wait for spontaneous resolution.

Another study^13^ has shown that a longer interval from the last menstrual period at the time of presentation is a predictive factor for spontaneous resolution of ectopic pregnancy. Ectopic pregnancies in which the last mens trual period was more than 6.5 weeks before the time of presentation were found to be 7.4 times more likely to undergo spontaneous resolution. Those authors found that 42% of ectopic pregnancies with a low initial beta-hCG level were spontaneously resolved. Their finding may also suggest that ectopic pregnancies detected or presented later are more likely to resolve spontaneously. They tried to explain their finding by postulating that it might be a self-selection phenomenon, in that ectopic pregnancies that are not treated early may outgrow their blood supply. They also demons trated that a lower initial beta-hCG level was a significant predictor for spontaneous resolution of ectopic pregnancy.

The findings from the present study demons trated that the early diagnoses were made among patients with fewer days of amenorrhea (6.81 ± 1.88 weeks) and higher beta-hCG levels (2642.7 ± 2315.1 mIU/ml), and the late diagnoses were made among patients with longer amenorrhea duration (8.87 ± 1.71 weeks) and lower beta-hCG levels (648.8 ± 754.7 mIU/ml). The success rate among the patients treated with methotrexate was 75.4%, compared with 95.4% for expectant management. Thus, the treatment failure rate in the methotrexate group was higher, which demonstrates the aggressiveness of the ectopic pregnancies in this group. Despite receiving medical treatment, these patients’ ectopic pregnancies continued to grow until rupture.

One interesting feature of the medical treatment was the follow-up after the single dose of methotrexate. The successful cases may have demonstrated the natural evolution of tubal pregnancy without surgery. Knowledge of this evolution facilitates the understanding of expectant management. After these patients received the single dose of methotrexate, they were evaluated by means of serial assaying of beta-hCG levels until the titers became negative. The usual length of time for the levels to become negative through medical treatment is around four weeks.^9,14^ In the present study, the gestational age for starting the medical treatment was 6.81 ± 1.88 weeks. Thus, the mean length of time in this study for the titers to become negative was 11.0 ± 2.0 weeks from the last menstrual period. Comparing these results with the expectant management in the present study provides an explanation for why the interval from the last menstrual period was longer in this situation (8.87 ± 1.71 weeks). Usually the time until tubal rupture is around six to eight weeks. If the process evolves beyond this period, the risk of rupture is almost over. In this situation, the beta-hCG titers decline, and the length of time since the last menstrual period is longer, thus demonstrating an old ectopic pregnancy (chronic case).

Another important point regarding medical treatment^15,16^ is that the beta-hCG titers can become negative before the ultrasound image disappears. Some authors have demonstrated that the length of time until the image disappears on ultrasound is 20 to 147 days (mean: 83 days), following medical treatment with methotrexate.^15^ In the present study, it was observed that the majority of the cases with expectant management were diagnosed later (8.87 ± 1.71 weeks), thus demonstrating that in this group of patients the risk of rupture had passed by and that the natural evolution was spontaneous resolution of the tubal pregnancy.

Importantly, long-term follow-up of women treated with methotrexate for gestational trophoblastic disease has not shown any increase in congenital malformation, spontaneous abortion, or second tumors following chemotherapy. Thus, none would be expected after treatment for ectopic pregnancy, because a smaller total dose is required and shorter treatment duration is used.^17-19^

There were some limitations to this study. In ectopic pregnancies with clinical treatment, no surgical proof was obtained. Therefore, some ectopic pregnancies that spontaneously resolved could have theoretically represented failed intrauterine pregnancy with trophoblasts undergoing regression. Considering the reportedly high specificity of the presence of an extraovarian adnexal mass for diagnosing ectopic pregnancy,^3^ this is probably a rare phenomenon. Another problem is that some ectopic pregnancies that are considered for expectant management on the basis of a drop in the patient's beta-hCG level require treatment because of subsequent increase in beta-hCG levels or increasing symptoms. Although this is rare, there is a risk of rupture of ectopic pregnancy during follow-up even if the titers of beta-hCG decline.^20^

## CONCLUSIONS

Women diagnosed with ectopic pregnancy may be categorized into two groups: those with an early diagnosis, and those with a late diagnosis. Early diagnosis is characterized by a shorter time since the last menstrual period, serum beta-hCG levels increasing or maintained over 24 and 48-hour intervals, higher beta-hCG levels, rapid growth, and higher probability of tubal rupture. Therefore, these cases require medical treatment with methotrexate or surgery. Late diagnosis is characterized by a longer time since the last menstrual period, beta-hCG levels decreasing over 24 and 48-hour intervals, lower beta-hCG levels, a latent prolonged clinical course, and lower chance of tubal rupture. Therefore, these cases require expectant management.
